# Innovating to enhance clinical data management using non-commercial and open source solutions across a multi-center network supporting inpatient pediatric care and research in Kenya

**DOI:** 10.1093/jamia/ocv028

**Published:** 2015-06-10

**Authors:** Timothy Tuti, Michael Bitok, Chris Paton, Boniface Makone, Lucas Malla, Naomi Muinga, David Gathara, Mike English

**Affiliations:** ^1^ KEMRI-Wellcome Trust Research Programme, P. O. Box 43640 - 00100, Nairobi, Kenya; ^2^ Nuffield Department of Medicine, University of Oxford John Radcliffe Hospital, Headington, Oxford, OX3 9DU, UK

**Keywords:** clinical data management, open source, clinical research, quality assurance, metaprogramming

## Abstract

**Objective** To share approaches and innovations adopted to deliver a relatively inexpensive clinical data management (CDM) framework within a low-income setting that aims to deliver quality pediatric data useful for supporting research, strengthening the information culture and informing improvement efforts in local clinical practice.

**Materials and methods** The authors implemented a CDM framework to support a Clinical Information Network (CIN) using Research Electronic Data Capture (REDCap), a noncommercial software solution designed for rapid development and deployment of electronic data capture tools. It was used for collection of standardized data from case records of multiple hospitals’ pediatric wards. R, an open-source statistical language, was used for data quality enhancement, analysis, and report generation for the hospitals.

**Results** In the first year of CIN, the authors have developed innovative solutions to support the implementation of a secure, rapid pediatric data collection system spanning 14 hospital sites with stringent data quality checks. Data have been collated on over 37 000 admission episodes, with considerable improvement in clinical documentation of admissions observed. Using meta-programming techniques in R, coupled with branching logic, randomization, data lookup, and Application Programming Interface (API) features offered by REDCap, CDM tasks were configured and automated to ensure quality data was delivered for clinical improvement and research use.

**Conclusion** A low-cost clinically focused but geographically dispersed quality CDM (Clinical Data Management) in a long-term, multi-site, and real world context can be achieved and sustained and challenges can be overcome through thoughtful design and implementation of open-source tools for handling data and supporting research.

## INTRODUCTION

The Kenya Medical Research Institute (KEMRI)-Wellcome Trust Research Programme’s Health Services Unit has engaged in a number of projects with the Kenyan Ministry of Health (MoH) since 2002 with work spanning evidence synthesis to develop national evidence-based clinical guidelines for pediatric care ^1^ , conducting pragmatic clinical trials, and surveys of the quality of care within hospitals ^2^ (KEMRI serves the same function as Medical Research Council in the United Kingdom and Center for Disease Control in the United States). This has helped evolve a unique platform of work focusing on implementing key interventions in Kenyan hospitals to improve the delivery of pediatric care across the country, the most recent of which is the Clinical Information Network (CIN). The CIN comprises a partnership between KEMRI, the MoH, The Kenya Paediatric Association, and 14 district-level hospitals that are spread out across the country. The aim of the network is to collect standardized routine data on pediatric admissions that will provide the basis for promoting adoption of evidence-based interventions, improving quality of care and, ultimately, support for pragmatic intervention trials designed with all stakeholders. CIN collects pediatric clinical data abstracted from medical records and data is in the domains of history, examination, laboratory investigations, diagnosis, treatment, and supportive care. A shared, standard pediatric admission record form developed in Kenya ^3^ and adopted by the MoH linked to a set of national clinical guidelines that define key symptoms, signs, illness definitions, and treatment strategies for the most common conditions provide the basis for core elements of the clinical process on admission The CIN focuses on these data, admission treatment, and investigations and discharge status. The design of the standard pediatric admission paper record was purposefully structured to focus on key clinical features central to care of common illnesses that are typically captured using binary or categorical fields (checkboxes, yes-no options).

Good clinical data management (CDM), also referred to as Data Quality Management, is the cornerstone of all these activities. For initiatives in low-resource settings such as this one, we argue that quality CDM in a long-term, multi-site, and real-world context can be achieved and sustained, and that challenges can be overcome by working closely with clinical teams and through thoughtful design and implementation of open-source tools for handling data. This paper outlines the approaches and innovations adopted to deliver good CDM within the CIN. Work with clinical teams that provides the basis for the network has been described elsewhere.

## BACKGROUND AND SIGNIFICANCE

There is increasing recognition of the need for improving the quality of care and pragmatic research to evaluate the effectiveness of interventions in real-life practice as an essential component of evidence-informed policy making. A key component of both is the quality of data ^4^ . The aim of the CIN is to work within, and not parallel to, existing health systems and with the healthcare workers that provide routine care. Source documents for research must therefore be those used in practice rather than being specific study case report forms typically completed by an entirely separate study team. To achieve this goal a number of challenges must be overcome. These include:

1. Resource context: There is shortage of staffing in a typical public hospital, and most public hospitals do not have an information technology (IT) department or officer, have limited funds to pay software license fees, and may still have frequent power blackouts.
2. IT tnfrastructure: Most public health facilities in Kenya (and in CIN) do not have comprehensive electronic health record (EHR) systems. National implementations of EHRs, like Kenya electronic medical records (EMRs), are generally limited to vertical programs dealing with Human Immunodeficiency Virus targeting outpatient visits and systems to computerize billing. There are very few cases of their use for inpatient care where there are major challenges in managing the complex longitudinal patient data ^5^ . The stability of internet connections is generally poor, varies based on the geographical location of the health facility, and hospital computers are often not connected to the internet via the hospital network.
3. Pre-existing reporting structures: existing national District Health Information System (DHIS) (requirements must be satisfied (the DHIS aims to serve some of the most basic functions of systems such as the Health and Social Care Information Centre in the United Kingdom and Healthcare Cost and Utilization Project in the United States). Careful attention must therefore be paid to stakeholder needs when redesigning traditional medical records so that they meet the needs of clinical users and the wider health information system. Research may also introduce new needs. Currently, the DHIS2 used in Kenya and widely in Africa as a national reporting framework captures no data on process of care or treatment from inpatient settings; these data are critical to the monitoring of medical interventions and quality of care within CIN and require the development of new data tools to collect them.
4. Data collection tools: Traditional paper medical records in routine settings rarely support good data collection as there is little standardization of the data model. EMRs are poorly developed and so have not (and are not at present designed to) promote such inpatient standardization in Kenya or much of Africa ^3^^,^^6^ . Work with the MoH and hospitals by the research team in the past has enabled production of an agreed, standardized medical record form that is a good fit to routine workflows and addresses some of these problems (described elsewhere) ^3^ . This can then become the basis for data capture either by abstraction to a data system (as we now describe) or in future might provide the common data model as part of an EMR.

In the past, different platforms had been used to manage clinical data within the research team. They included an MS Access solution, an in-house open source solution using PHP/MySQL and the ZEND Framework, and OpenClinica 3.0 community edition ^7^ .

1. The first transition was from MS Access to a bespoke PHP/MySQL solution. In this phase, HSU was trying to overcome the limit on the number of variables a project could have. Also, the team wished to limit the costs associated with proprietary software, particularly due to planned multi-site use in resource-limited public institutions.
2. The second transition was from the bespoke PHP/MySQL solution to OpenClinica. While flexible, the PHP/MySQL solution required constant redevelopment and upgrading to keep up with the ever-evolving the research team projects’ needs. It was also not designed to support the rigorous requirements of CDM for a clinical trial. OpenClinica was implemented for a pragmatic multi-site clinical trial carried out at KEMRI ^8^ but seemed over-elaborate for long-term observational studies.

The research team’s previous experience with OpenClinica demonstrated the advantages of its use of an excel sheet to design data collection tools that enabled full participation of clinicians in database design and ease of sharing meta-data. Exploring other open source tools known to the research team and colleagues with this feature we opted to explore the use of Research Electronic Data Capture (REDCap) that was designed for observational studies and for attributes described in detail in the methodology.

## OBJECTIVE

The research team therefore required a solution with the following characteristics: straightforward tool design and updating, accurate case-record capture (with flexible, well designed layouts), efficient and effective auto-collation of multi-site data, minimal cost of running the CDM solution, and that facilitated data quality assurance. This solution would have to implement data capture based on existing clinical tools while focusing on data required for practice adherent to the national evidence-based clinical guidelines for pediatric care ^9^^,^^10^ . The solution’s data entry component was not meant to be or function as an EMR.

## METHODOLOGY

To meet the afore mentioned needs and overcome challenges in Table 2 , we adopted the REDCap Programme in three initial observational studies and further optimized this tool for the current CIN project ( Table 1 ).

**Table 1:** Previous KEMRI – Wellcome Trust Programme’s projects and the respective CDM platforms/tools used

Project	Study Type	Platform	Database	Additional Useful Features
8 District Hospitals Study (2006–2008)	Observational	MS Access	MS Access 2005	
KNH Work (2008–2009)	Observational	Zend Framework (PHP)	MySQL	
Electronic Paediatric Admission Record (EPAR) (2009–2010)	Observational	Zend Framework (PHP)	MySQL	
Pneumonia Trial (2011–2013)	Clinical Trial	OpenClinica (JAVA)	PostgreSQL	
Pneumonia Observational Study (2012–2013)	Observational	REDCap (PHP)	MySQL	Web API
Health Services, Implementation, Research and Clinical Excellence (SIRCLE): 22 Hospital Survey (2012)	Observational	REDCap (PHP)	MySQL	Web API
GEF Surveys (Maternal, Neonatal, Pediatric) (2012–2014)	Observational	REDCap (PHP)	MySQL	Web API
Clinical Information Network - Ongoing (2013)	Observational	REDCap (PHP)	MySQL	Web API

REDCap = Research Electronic Data Capture; API = application programming interface

Each tool transition was aimed at trying to satisfy a specific set of CDM needs but each was associated with challenges as shown in Table 2 below, which prompted the search for a different solution for CDM across the CIN.

**Table 2:** Challenges of key data management platforms previously used in KEMRI Wellcome Trust and factors necessitating transition to a different CDM solution

Alternate Solutions Used and Challenges Leading Up to REDCap Selection	
MS Access	Double data entry of paper forms was cumbersome. It did not provide an opportunity to correct possible mistakes in the forms.
	Inability to create tools with more than 255 fields.
	Software is commercial: increased project costs due licenses for each study machine.
	Cumbersome to update data collection tool. Requires re-design the data collection tool from scratch.
	Ms Access at the time did not allow for a multi user interface to be created. Thus for several data entry clerks, each had to have access to local a dedicated copy of the database.
	Use of Ms Access required one to have simple database management skills.
In-house open source solution (Zend Framework based)	Manual data transfer and extraction for analysis in a multi-site environment.
	Updating the data collection tool still required personnel with specialized skills.
	Changes to the structure of the project required reprogramming of the PHP solution creating a lag in implementing updates within the required time frame.
OpenClinica 3.0 Community Edition	Initial tool development process was complicated.
	Training data clerks on how to use OpenClinica was a challenge.
	Lack of system upgrades, data entry rule designer feature, a data mart (OpenClinica’s feature: allows export of clinical data in a readily accessible flat file for reporting and analysis), system patches, support for automated validation and data quality management for community edition.
	Clinical trials oriented: may not be a good fit for observational studies.

REDCap = Research Electronic Data Capture.

REDCap is a novel workflow methodology and software solution designed for rapid development and deployment of electronic data capture tools to support clinical and translational research. The software was initially developed and deployed at Vanderbilt University ^11^ . It currently has collaborative support from a wide consortium of more than 1200 domestic and international partners, having deployed over 138 000 projects and over 188 000 users. REDCap has a simple enough interface for researchers to create data collection tools with minimal support from the data manager. This enables data managers to focus on optimizing data management procedures. REDCap was first installed ( Table 2 ) on 20 ultra-portable laptops for concurrent multi-site survey work with a synchronization module developed by the research team that allowed data to be transferred to a central server. Some of the general properties that were of key interest that were found in REDCap and that precluded the use of other popular open source solutions such as Epi Info and Epi Data included:

1. Low hardware specification and platform independence: REDCap is very lightweight with regards to processing power, memory or hard drive space and is compatible with all operating systems.
2. Secured and web-based: REDCap can be setup locally on a machine to allow data capture. When that machine is connected to a network/internet, it can be configured to securely transfer data to a remote REDCap installation. In settings where there is no internet connectivity, it allows offline data collection and use of an internet modem to send data to a remote REDCap installation periodically or when there is reliable connectivity. It supports secured web authentication, data logging, and secure sockets layer encryption.
3. Supports multi-site access: REDCap’s projects can be accessed by multiple users from multiple locations. Also, through an application programming interface (API), multiple remote instances can synchronize data to a central REDCap installation through the internet.
4. Allows fast and flexible development of data collection tools encompassing large numbers of variables and doesn’t require any technical skillset to implement.
5. Data management tools are fully customizable and allow for mid-study modifications without affecting previously collected data.

In response to the challenges outlined in the background section, a desktop computer running on Linux with R and REDCap installed, an internet modem, and an uninterrupted power supply (UPS) unit was provided for each hospital in CIN. These items would be serviced by the hospital’s IT or maintenance department in case of failure. The modem is used for sending data from sites that have poor or no internet connection. Because of the frequent unexpected power disruptions, the UPS protects the equipment against damage and data loss, and mitigates against disruption of data collection. A data clerk with health records information experience was seconded to the health records departments of all the CIN hospitals. They are co-supervised by both the hospital’s Health Records Information Officers and research data management officers.

### Clinical Information Network Data Collection

The standard clinical documentation records are structured ^12–15^ with the majority of the form providing binary or categorical options for clinicians to select. Patient treatment sheets and laboratory registers, additional source documents, are relatively standard across government hospitals. To guide collection of data comprehensive written guidance in the form of standard operating procedures is provided, a manual also used in primary training of clerks. This guidance and training have been refined during studies conducted over a number of years ^16–18^ enabling clerks to follow clear procedures to abstract data from the patient paper records directly into the electronic form with a minimum of interpretation required. The design of the CIN, however, provided a number of significant challenges for the research team. The CDM framework implemented to support CIN operates in the following way:

1. It provides support for daily CIN data collection by a single person with minimal training, although a supervisor would support them through telephone calls as required and visit every 2 months.
2. Based on the key fields of the patient record, the CDM framework provides for queueing of the record for full data or minimum data collection. When tagged for minimum data collection, only data required for HIS reporting would be collected. This allows focusing of data collection to specific clinical groups and permitted control of data capture workloads (see below).
3. As the data is being entered, The CDM should provide for synchronous data validation. The data clerk would receive message alerts for any input that was outside acceptable margins or did not conform to pre-specified CDM validation rules using pop-up messages. The CDM framework also provides support for both soft and hard error validation. One may override error alerts from soft validation while errors from hard validation would not allow data submission until they are resolved.
4. The CDM framework provides support for complementary data quality validation using R cleaning scripts (described below) run by the clerk at the end of each day as part of quality assurance measures. Execution of these scripts generates an error report, allowing the clerk to resolve any data discrepancies before data submission. These R scripts complement the data collection tool’s internal validation mechanisms by adding an extra layer of validation rules based on epidemiological constructs that are too complex to implement within the data collection tool—e.g., validation of classification of diagnosis, based on tuples of diagnoses the data clerk entered.
5. The framework provides web-based mechanisms to collate on KEMRI central servers anonymized data from all hospitals once the quality discrepancies had been resolved. At the data store, an automated R script provides secondary quality checks on the master data store, containing data from all sites and generates a daily error report. This report is used by the supervisor to provide daily feedback to data clerks at the site, allowing them to resolve further discrepancies observed if possible and resend the data. This serves as second line of quality verification.
6. The framework allows intermittent external data quality assurance exercises to be conducted bi-monthly by the supervisor who would undertake an independent, duplicate entry of a sample of records. Concordance evaluations are then carried out on the spot using R scripts to assess data collection accuracy. The results are then fed back to the clerk to ensure high quality of data is maintained.

**Figure 1:**
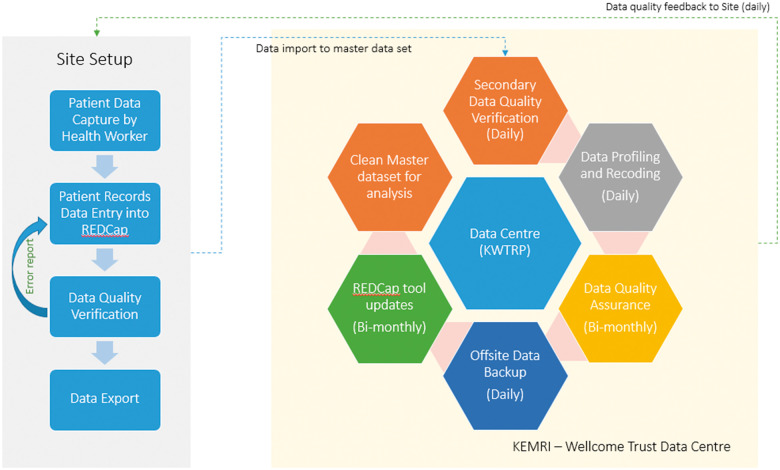
**CIN's data management framework and workflow nature** . Data entry into collection tool is after patient death/discharge.

Once the quality has been verified (after step 5 above), the data is profiled, coded and backed up onto a remote server. A clean master dataset is thus produced for analysis by statisticians and epidemiologists (see Figure 1 below).

**Figure 2:**
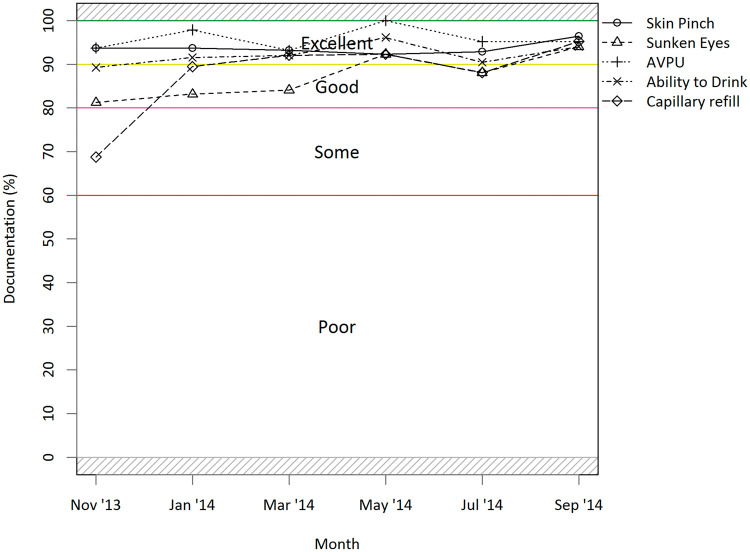
Dehydration indicator documentation trends reflect level of documentation of five key clinical signs of all dehydration cases as recommended for assessment of severity and treatment according to national guidelines. Figure is from a routine hospital report now provided to CIN hospitals.

## RESULTS

### Key features and innovations supporting data collection

#### 1. Randomization and branching logic

REDCap’s inbuilt randomization feature allows the technical team to be able to randomly select pre-specified fields for data collection. This feature has been activated for hospitals with extremely high workload, where patients being discharged are 2–3 times as many as the CIN daily discharge average for hospitals. Using this feature, the decision to collect a minimum dataset (180 variables) required for standard health information system reports or a full dataset (367 variables) on each new record being entered is decided at random ensuring a representative sample of records have a full set of data collected. This procedure reduces the time a clerk spends collecting data by half if the minimum dataset is being collected. This then allows us to balance workloads of different sites by varying the proportion of case records selected for full or minimum data collection while ensuring critical data is collected on all.

Branching (decision tree) logic is used to implement skip patterns in which data entered with values equal to predefined expressions triggers subsequent field selection/deselection in the same record. This feature allows researchers to map out a data collection path based on clinical pathways, depending on what clinical data the clerk was entering. It also allows us to manage data collection during leave periods, hospital strike periods and data collection for nonmedical pediatric admissions while guaranteeing that the minimum dataset per record required for health information system reporting is captured. For example, when dates of discharge fall between holiday periods, or when collecting data on a surgical patient, this feature can be used to require data collection for only the minimum dataset while hiding nonessential or irrelevant data fields.

#### 2. Data lookup feature

In the initial version of REDCap deployed to hospitals only a set of 18 common diagnoses were precoded to support structured input. As the nature and naming convention commonly used was not known at that stage, less common diagnoses were entered as free text. For similar reasons free-text entry was initially employed to capture data on use of less common drugs. After 6 months, a review of the data collected as free text was undertaken and used to inform the development of a plugin module for REDCap to allow for selection of pre-specified, standardly named responses (e.g., drug names) using a look-up table with auto-complete functionality. In the same way, we have been able to introduce the International Classification of Diseases (ICD-10) ^19^ coding for diagnosis fields. This latter development enables the system to provide routine health information reports to the hospital records office. These lookup features support automation of the coding of diagnosis to ICD-10 and of treatment data to generic drug names. The clerk uses the drop-down to select values from a list of entry options that are labeled based on local terms but stored as standardized nomenclature.

#### 3. Application Programming Interface

REDCap is a secure web application supporting Lightweight Directory Access Protocol (LDAP) authentication, data logging, data access groups and Secure Sockets Layer (SSL) encryption of data. Through REDCap’s web API (with a token hash representing the API project, username, and password), data can be pulled from a REDCap project and pushed to another REDCap setup or into a statistical environment for analysis. In our setting, the web API is used together with a synchronization module developed in PHP, JQuery and MySQL to push data collected in the field to the master dataset at the program’s servers. This ensures that at the end of every day the researchers have all the data that is at all sites giving us an opportunity to monitor data collection progress and data quality. In addition, the web API allows researchers to program R cleaning scripts to pull data from the REDCap setup in the local machine or a remote server into the R environment and perform quality analysis on the data and generate error reports at the end of each day. The clerk can then take action on the reports using prespecified guidelines in the standard operating procedures for data collection for the project.

Initially, the synchronization module utilizing REDCap’s web API worked well in collating the data in multiple sites for multiple projects but with the increased data transferred and with the large number of data entry fields for the project (367 per patient), the synchronization module ^20^ started to fail. With every synchronization request, the approach resulted in the entire data from the beginning of the project being sent. This caused a failure of synchronization because of limits in script execution time and poor internet connectivity in some regions. The research team therefore customized the requests made to REDCap’s web API by adding the ability to limit data to synchronize by date (selected by the clerk with default set to last 30 days) to resolve synchronization issues.

#### 4. Data checking and progression to meta-programming

In the early stages of using REDCap together with R, data quality control was through a manual approach. In this early approach, R cleaning scripts would be manually programmed to implement validation logic in REDCap. Data would then be downloaded from a REDCap project as a Comma Separated Values (CSV) file to a prespecified folder on the local machine and the R scripts would be executed to generate an error report. The data clerk in this early approach would then use the error reports to resolve any data discrepancy issues after which, he/she would securely compress the CSV data file and send it to the research team by email. In an update to the approach, the team now uses the REDCap’s web API to pull the metadata (data about a specific project’s individual variable names, variable types, structure and flow, variable validation rules) into R’s environment. From the metadata, R is able to auto-generate code and scripts that are used in data quality validation and hospital reporting and execute them. This in-turn delinks changes attributed to updates in the data collection tool to version specific quality validation procedures written in R and automates propagation of validation rules from REDCap as it is updated into R cleaning and reporting scripts. A summary of challenges encountered and some implemented and longer term solutions are detailed in Table 3 .

**Table 3:** Summary of challenges encountered in implementing the Clinical Data Management (CDM) framework for CIN and solutions developed (normal text) or proposed (italicized text).

Challenge	Details of Challenge	Long-term Solution
Resource limitations	Internet connection challenges, staffing challenges, insufficient computers, power outages, software licensing costs	1. Hire a second clerk to each CIN hospital to support the network 2. Provide internet modem to all clerks to use to send data 3. Provide a computer to each hospital for data capture 4. Use open-source /freeware tools to support data capture—i.e., machine running on Ubuntu, REDCap for data capture, R for analysis 5. Use UPS units to mitigate effects of power outages
Adoption of data codification standards	Use standard nomenclature to code variables used in pediatric data at point of data transfer from paper to electronic form	1. Use lookup lists for: – Treatment fields to capture store generic drug name when brand name entered – Diagnosis fields to capture values as their ICD-10 equivalent 2. Implement SNOMED-CT equivalent for all CIN variables and make the code book available when sharing data 3. Collaboration with Ministry of Health and Kenya Paediatric Association, hospital clinical staff to adopt a standard clinical data model for pediatric patients tied to SNOMED-CT / ICD-10
Data synchronization	Automate data consolidation	1. Update synchronization module to allow data export in batches 2. Set the maximum limit of data transfer to observations not older than 30 days
Data quality control	Ensuring good quality data is being captured	1. Create standard operating procedures for data collection 2. Use cleaning scripts to validate accuracy of data entered at the hospital level 3. Conduct bimonthly data quality assurance exercise, using a sample of previously entered records 4. Generate daily data quality reports and use them to go through any errors with data clerk over a telephone call
Data collection tool creation and update	Integrate CIN data collection with routine job aides and work flows, allow for updates	1. Design data abstraction of paper records to REDCap tool to reflect CIN hospital workflow and to ensure data elements needed for DHIS reports included 2. Data entry from paper records to REDCap tool to happen after patient discharge/death 3. Update REDCap tool the same time as when conducting bimonthly data quality assurance exercise
DHIS Reports for Ministry of Health	Generate and submit DHIS reports for each CIN hospitals directly to Ministry of Health	1. Create Script to Generate DHIS reports based on pre-specified cohort groups for pediatric care 2. *Engage the Ministry of Health to allow automated submission of DHIS reports to the national HIS through the web API* —this is still ongoing

API = application programming interface

## DISCUSSION

We required a good CDM framework that was relatively easy to setup and deploy rapidly, provided ease of use in resource-limited settings by clerical staff with minimal training and that supports routine information system needs and research. When the research team was designing the new CDM framework, development efforts were iterative and involved input from technical support personnel, epidemiologists, statisticians, and healthcare providers. This approach and skillset ensured a dynamic CDM framework would be implemented that would reflect current clinical practices, deliver quality data for research, and meet the needs of hospitals involved in the study ^21^^,^^22^ . Data collection was designed around hospital workflow, and happens after patient discharge or death. It was purposefully implemented to be versatile based on current hospital practices especially in areas around patient discharge and death.

The research team has been involved in prior work on developing paper based tools and improving clinical documentation in partnership with Kenya’s MoH that facilitates the data collection described ^3^^,^^9^ . This element—working with clinicians to agree core components of good medical records—needs to be appreciated as replication elsewhere would need to also focus on such clinical components. Such clinical engagement also lays a foundation for development of EHRs that provide a common data platform that is meaningful to clinicians and that ultimately might be used to produce better information ^23–25^ . There are limitations to extrapolating our experience to development of EHRs which are a considerably greater task and were beyond the scope of this project but lessons learned about how data can be used locally should inform such developments.

In the implementation of the CDM framework, data clerks’ experience using electronic data collection tools was revealed to be a relevant factor and augmented by purposefully developed, comprehensive standard operation procedures, and an intensive 5-day training period that ensured quality data is collected ^21^ efficiently. This helped improve the quality of data capture. The data validation refereed to here is in reference to standard operating procedures for translating the paper record into electronic form, not the accuracy of clinician’s documentation.

The technical team did not undertake a comprehensive review of open source solutions before opting for REDCap as the tool of choice for the CDM described. In using REDCap the technical team has, however, been actively involved in, and benefited from, REDCap forums and other developer communities. This has allowed us to contribute to improvement and extension of REDCap features. Such activities resulted in solutions such as the data lookups and development of the synchronization module that augment REDCap. Consequently, we were better able to support structured data collection and limit the need for free text data entry ^26^ . When designing the synchronization module, due consideration had to be given to the limited technology infrastructure at study sites as it had an impact on data export activities. Other innovations have included an ability to randomly select records after a unique identifier has been created for different levels of data entry allowing a single clerk per hospital to capture representative data in high volume settings admitting over 4000 children per year.

The CDM framework was aimed at avoiding creation of parallel structures, common in vertical programs in low-income settings, and had to meet mandatory reporting requirements of the MoH ^24^^,^^27^ . In addition, because previous studies have shown most routinely collected data to be poor, it was important to introduce robust data quality assurance measures. This was achieved by innovative use of the open source statistical package R integrated with REDCap using statistical programming approaches to ensure the quality of health data collected in terms of completeness, correctness, and consistency ^27–29^ .

Key challenges that remain ( Table 3 ) include the lack of a facility for remotely updating REDCap tools at each location and the inability to programmatically push routine hospital reports into Kenya’s national DHIS2 ^30^ implementation. This is because it has been configured to allow data submission only through online forms and file uploads while DHIS’s web API is disabled in Kenya’s national DHIS instance. The technical team also experienced difficulties integrating complex R computational models when running data analysis. R’s support for using multi-core systems and distributed computing is still insufficient for the needs of the project and, as the data produced grows, R’s ability to compute these analyses efficiently should grow with it. As it stands, we are exploring mechanisms of running the analysis using tools capable of complex computation in a big data environment such as Python ^31^ .

Longer term challenges include efforts within CIN to utilize standard nomenclature (e.g., Systematized Nomenclature of Medicine-Clinical Terms) for electronic data capture partly due to the lack of a detailed framework outside HIV programs to guide this at the national level in Kenya. Careful consideration given to design and use of standard terminology is particularly important in settings such as Kenya where EHR systems are beginning to emerge in a variety of forms and in geographically dispersed locations. Although there are at present no major legacy hospital EMR systems providing an opportunity to implement interoperability standards, this opportunity will be lost if delays result in the growth and spread of systems that do not have interoperability designed in. Careful attention now needs to be given to ensuring the development of interoperable EHRs that will support national health information requirements for routine system performance evaluation and research. Thus in most low-income settings no routine systems exist for regular monitoring of even the most basic aspects of inpatient quality of care, for example case specific inpatient mortality rates. In the research arena single site studies are the norm and data systems are typically withdrawn at the close of the study so that results may be hard to generalize while large scale health service evaluation research is largely absent.

At the heart of the data CIN collects are agreed good clinical practices that include a good quality initial patient evaluation—a process that was extensively tested with clinicians in prior work—linked to consensus developed national guidelines ^10^ . This work could contribute to the further development of the common data dictionary in KenyaEMR in the focus area of inpatient pediatrics and may provide a model for other inpatient clinical environments. We are in the process of contributing to consensus on SNOMED-CT archetypes for pediatric data, in partnership with local EHRs providers and pediatricians across CIN. This engagement also guides the recoding of medical data from local verbiage to standardized nomenclature and is inclusive of each individual hospital’s context (type of tests offered, equipment available, local pattern of disease, etc.). The initial work is focusing on patient history, examination, treatment and laboratory investigation data. Diagnosis data has already been coded using ICD-10. Such efforts will allow a copy of the original dataset to be kept with the agreed-upon codes applied onto the original dataset programmatically, resulting in a transformed dataset with standardized nomenclature being generated. Feedback on comparisons between the original dataset and the transformed data are evaluated recursively by the involved parties (pediatricians, health records information officers, health researchers, EHRs developers) until consensus is reached on the final dataset to be used.

## CONCLUSION

The demand for data has never been greater. The expectations are even greater for data to be used in conducting locally applicable research and supporting quality and service delivery improvements. In low-income settings there is considerable pressure to contain the cost of data acquisition and still implement effective data management frameworks that produce quality data. These frameworks need to be inherently scalable, capable of handling, and sending large volumes of data efficiently and affordably even in areas with poor unreliable internet connectivity. This paper has set out to show how HSU is responding to this challenge to support high quality clinical observational studies and quality improvement using well-designed data management frameworks that utilize open source tools in the face of the challenges that exist in much of Africa.

## Competing interests

None.

## Contributors

The roles of the contributors were as follows: T.T., and M.B. drafted with the help of M.E. an initial version of this manuscript. All authors contributed to subsequent drafts and approved a final version of this paper.

The Clinical Information Network members who contributed to the conduct of the work, collection of data, data quality assurance and development of data management and reporting frameworks that form the basis of this report include: Wycliffe Nyachiro, George Mbevi, and Morris Ogero.

## Funding

Funds from The Wellcome Trust (#097170) awarded to ME support T.T., MB, L.M., B.M., N.M. and D.G. Additional funds from a Wellcome Trust core grant awarded to the KEMRI-Wellcome Trust Research Programme (#092654) supported this work. The funders had no role in drafting or submitting this manuscript.
